# Supplementary material for: Enhancing sorption kinetics by oriented and single crystalline array-structured ZSM-5 film on monoliths
Source: Nat Commun. 2024 Jul 2;15:5541. doi: 10.1038/s41467-024-49672-5 (PMC11220059; doi:10.1038/s41467-024-49672-5)
Supplement: Supplementary file 1 — Supplementary Information [file 41467_2024_49672_MOESM1_ESM.pdf]

Supplementary Information

Enhancing Sorption Kinetics by Oriented and Single Crystalline Array-structured ZSM-5 Film on Monoliths

Junfei Weng[1], Chunxiang Zhu[1], Binchao Zhao[1], Wenxiang Tang[1], Xingxu Lu[1], Fangyuan Liu[1], Mudi Wu[1], Yong Ding[2], and Pu-Xian Gao [1,*]

[1]Department of Materials Science and Engineering & Institute of Materials Science, University of Connecticut, Storrs, Connecticut 06269, USA.

[2]School of Materials Science and Engineering, Georgia Institute of Technology, Atlanta, Georgia 30332, USA.

[*] e-mail: puxian.gao@uconn.edu.

- **Supplementary Figures 1-16**
- **Supplementary Tables 1-3**
- **Supplementary References**

Supplementary Figures

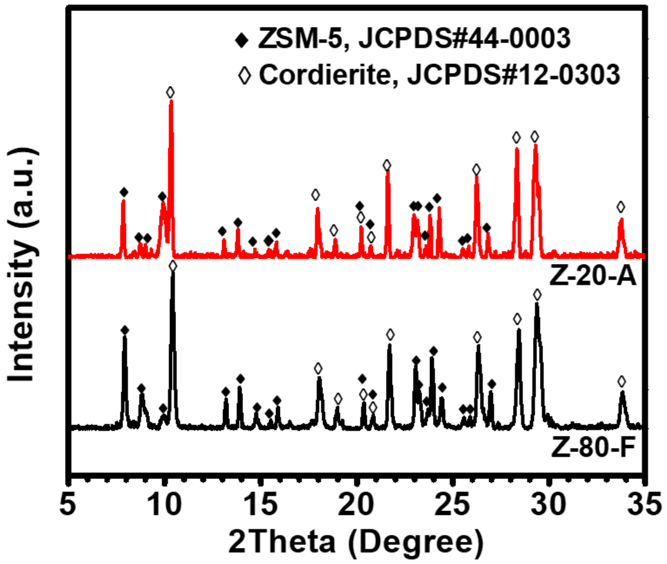

Supplementary Figure 1. X-ray diffraction patterns of the conventional continuous ZSM-5 film (Z-80-F, black line) and array-structured ZSM-5 film (Z-20-A, red line). Source data are provided as a Source Data file.

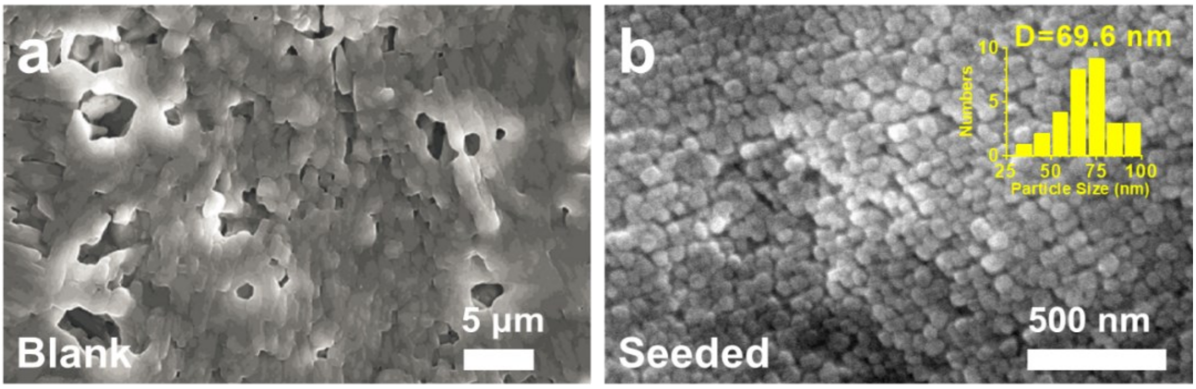

Supplementary Figure 2. Top-view scanning electron microscope (SEM) images. a blank cordierite. **b** the channel surface of cordierite monolith is covered by silicalite-1 seeds in a diameter of ~70 nm. Inset: particle size distribution of silicalite-1 seeds on channel surface.

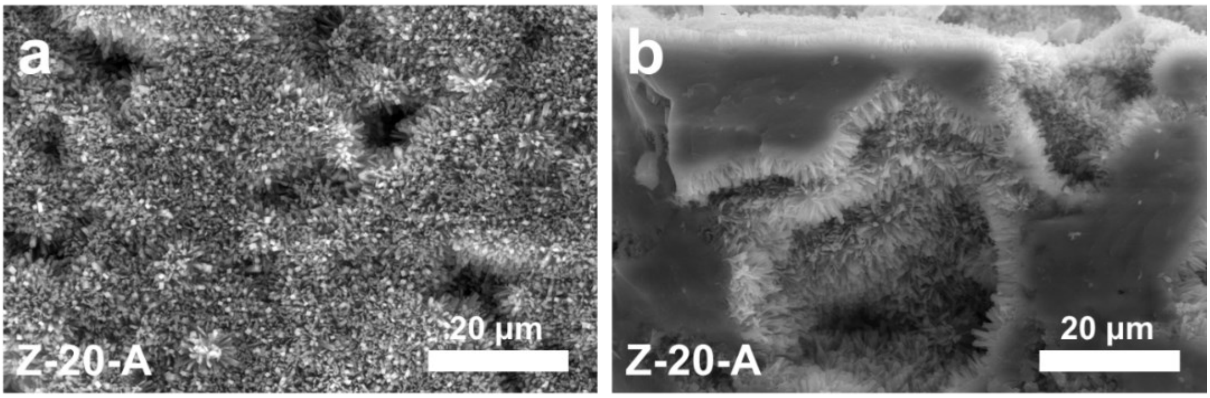

Supplementary Figure 3. Scanning electron microscope (SEM) images of array-structured ZSM-5 film (Z-20-A). a Top view. **b** Cross-sectional view.

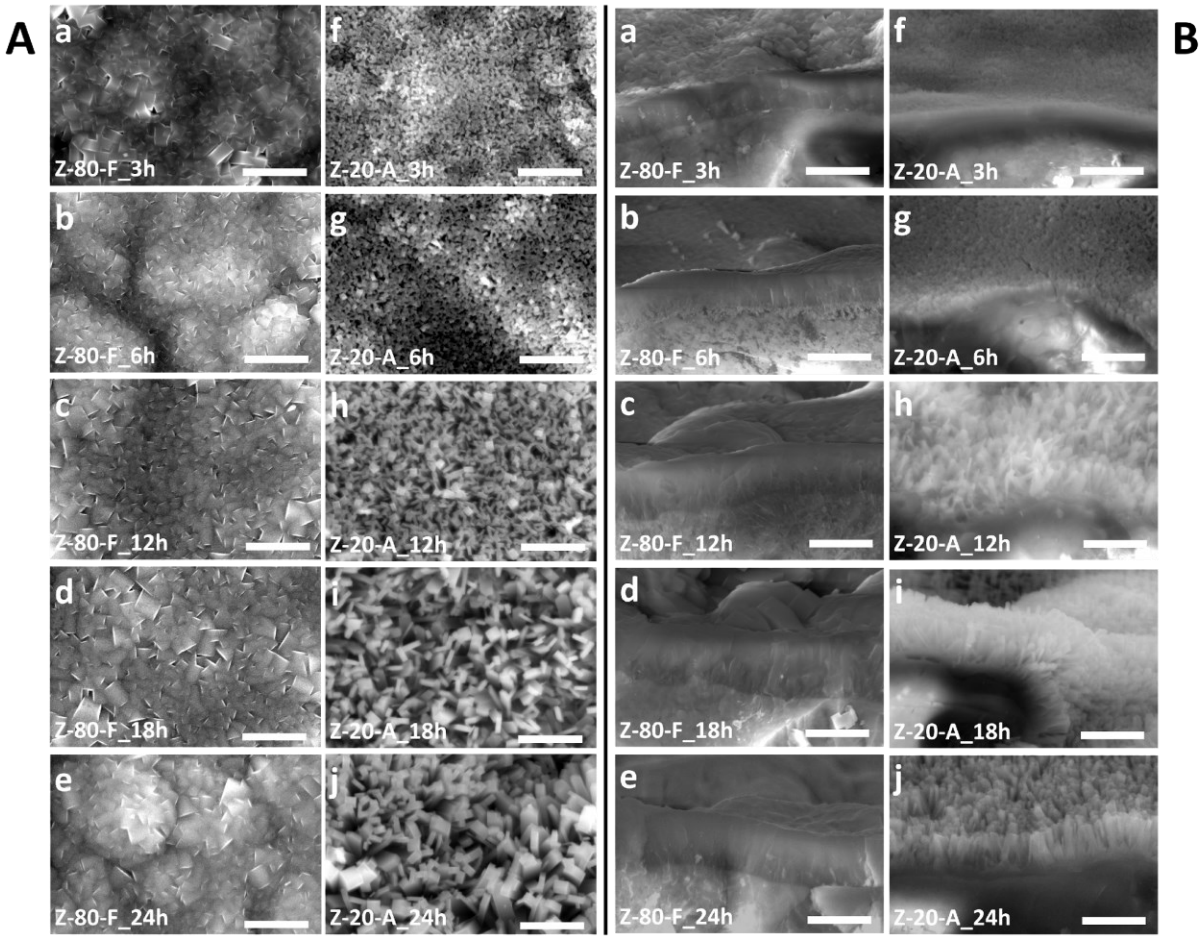

Supplementary Figure 4. Scanning electron microscope (SEM) images of two ZSM-5 film samples synthesized at 180 °C for 3, 6, 12, 18 and 24 h. **A** Top view. **B** cross-sectional. **a-e** Conventional continuous ZSM-5 film (Z-80-F). **f-j** Array-structured ZSM-5 film (Z-20-A). Scale bars: 5 µm.

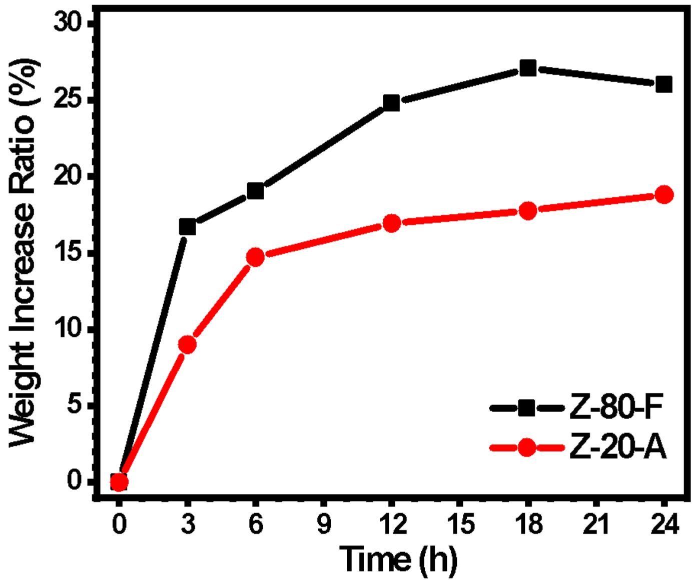

Supplementary Figure 5. Weight increase ratio of conventional ZSM-5 film (Z-80-F, black line) and array-structured ZSM-5 film (Z-20-A, red line) synthesized at 180 °C. Source data are provided as a Source Data file.

Growth of ZSM-5 films in Z-80-F and Z-20-A:

In general, the growth of zeolite film on the substrates with the help of seeds follows a competitive growth model, namely "evolutionary selection"[1, 2]. Specifically, pre-coated seeds will firstly grow larger to form an initial continuous film. As the growth proceeds, crystals are overgrown by adjacent ones, and grains with a faster growth rate will grow faster and gradually bury more slowly growing neighboring grains. For MFI crystals, c-orientation usually grows fastest, followed by b-orientation and then a-orientation. As a result, ZSM-5 films usually end up with a c-oriented columnar structure given the sufficient crystallization time.

As revealed in Supplementary Figure 4, upon contacting the synthesis solution for Z-80-F, the silicalite-1 seeds quickly develop into a continuous ZSM-5 film with intergrown crystals at first 3 h. The intergrowth of ZSM-5 crystals exists throughout the whole synthesis process, resulting in a thickness of 4 μm in Z-80-F associated with a total loading ratio of 26.0 wt.% after 24 h. Comparably, for the array-structured film, ZSM-5 crystals do not intergrow with each other even at the initial stage, leaving spaces between crystals to help maintain the crystal individuality. The morphology of ZSM-5 nanorods becomes visible after 6 h, and finally, an array-structured film is formed with ~3 μm in thickness and 19.2 wt.% in total loading ratio (Supplementary Figure 5).

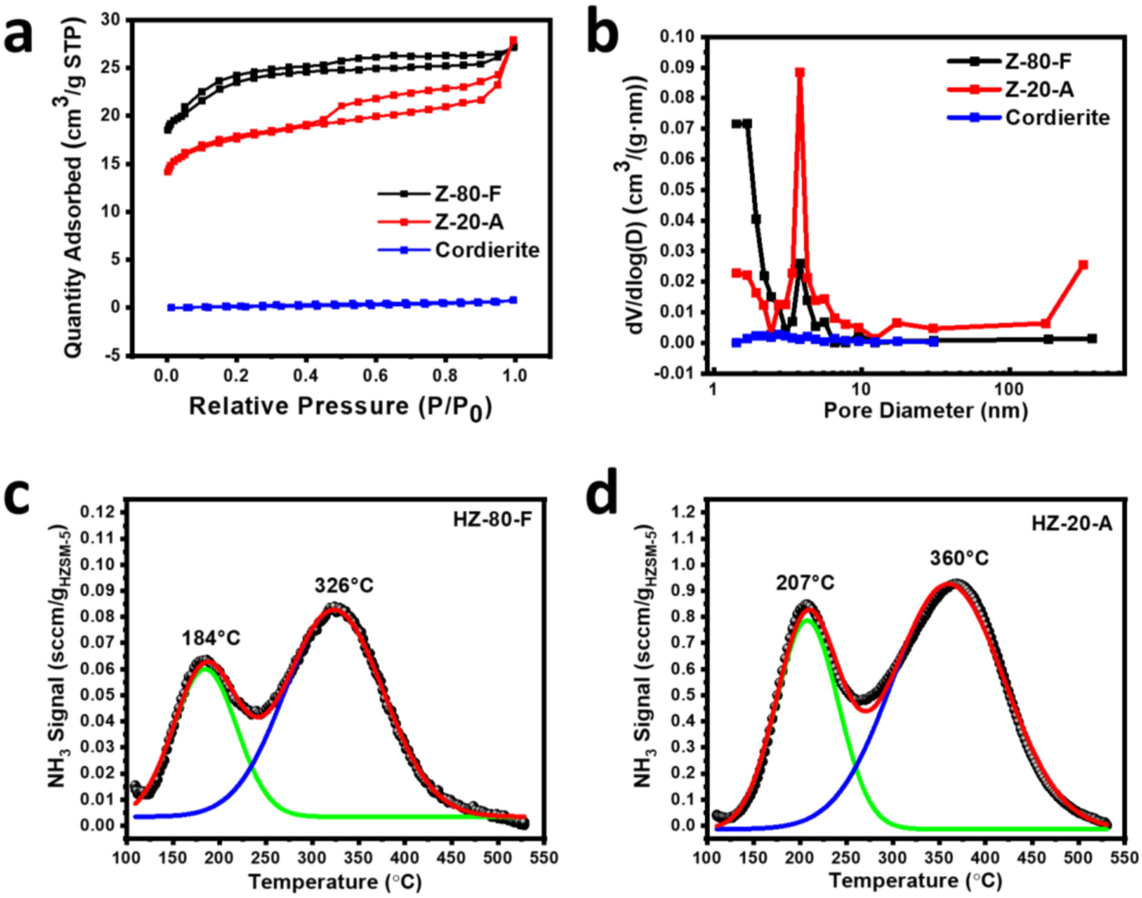

Supplementary Figure 6. Physicochemical properties of the as-prepared conventional ZSM-5 film (Z-80-F, black line) and array-structured ZSM-5 film (Z-20-A, red line). a Nitrogen adsorption-desorption isotherms. **b** Pore size distribution derived by BJH method. The results from cordierite substrate (blue line) were also included. **c** Ammonia temperature-programmed desorption (NH3-TPD) of HZ-80-F. **d** NH3-TPD of HZ-20-A. The green and blue line were the corresponding deconvoluted results. Source data are provided as a Source Data file.

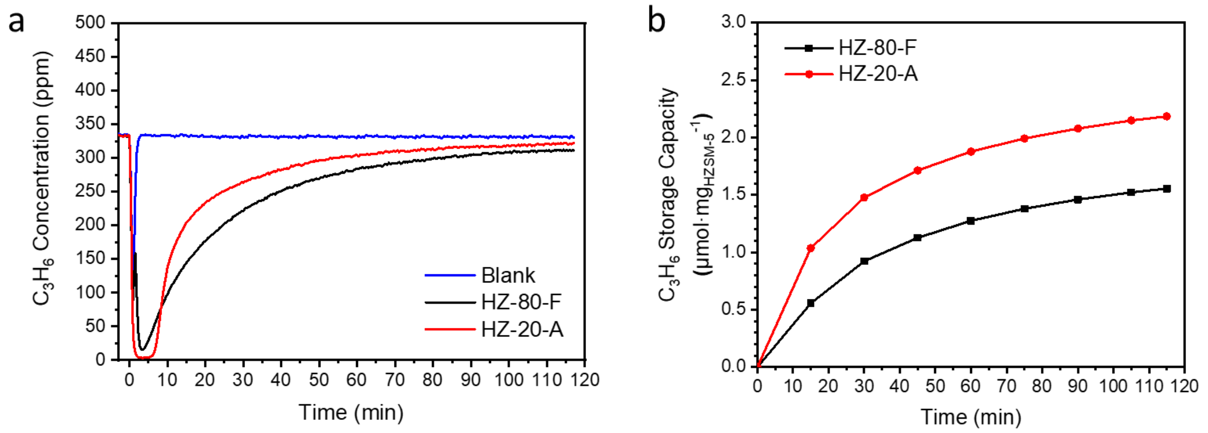

Supplementary Figure 7. Breakthrough test of propene adsorption on HZ-80-F (black line) and HZ-20-A (red line) at 100°C for 120 min under the feeding gas of 333 ppm C_3H_6 + 12 % O_2 / N_2. a Adsorption behaviors of propene concentration change vs. time. The result of blank cordierite substrate (blue line) was included. **b** Calculated average propene storage capacity normalized by the tested H-ZSM-5 mass within 120 min adsorption. Source data are provided as a Source Data file.

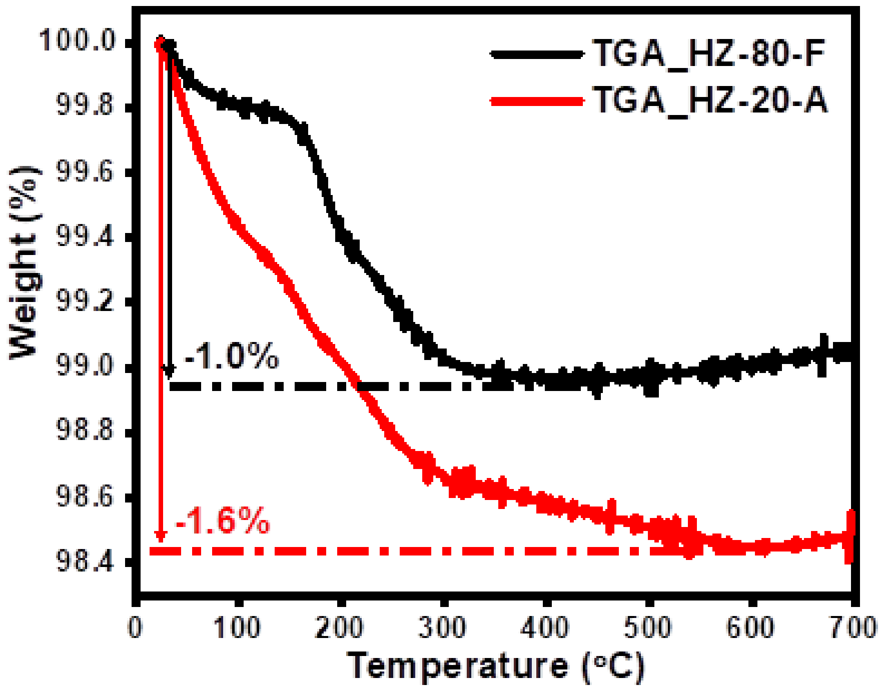

Supplementary Figure 8. Thermogravimetric analysis (TGA) results of HZ-80-F and HZ-20-A after the adsorption under 333 ppm C_3H_6 + 12 % O_2 / N_2 at 100 °C for 30 min. Source data are provided as a Source Data file.

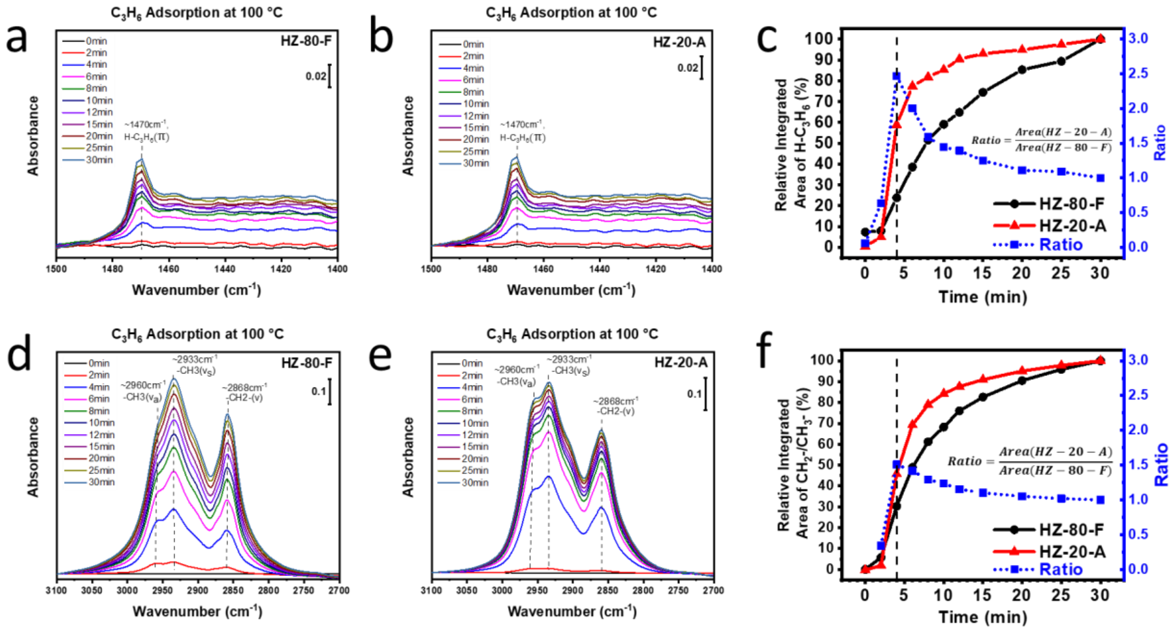

Supplementary Figure 9. *In situ* diffuse reflectance infrared Fourier transform spectroscopy (DRIFTS) spectra of two proton-exchanged ZSM-5 film samples during the propene adsorption under 333 ppm C_3H_6 + 12 % O_2 / N_2 at 100 °C for 30 min. a, d DRIFTS spectra at 1500-1400 and 3100-2700 cm^{-1} of proton-exchanged conventional ZSM-5 film (HZ-80-F), respectively. **b, e** DRIFTS spectra at 1500-1400 and 3100-2700 cm^{-1} of proton-exchanged array-structured ZSM-5 film (HZ-20-A), respectively. **c, f** The relative change of the integrated peak area at H-bonded C_3H_6 (1470 cm^{-1}) and CH_2-/CH_3- (3080-2750 cm^{-1}) from DRIFTS spectra during the propene adsorption at 100 °C for 30 min, respectively. Source data are provided as a Source Data file.

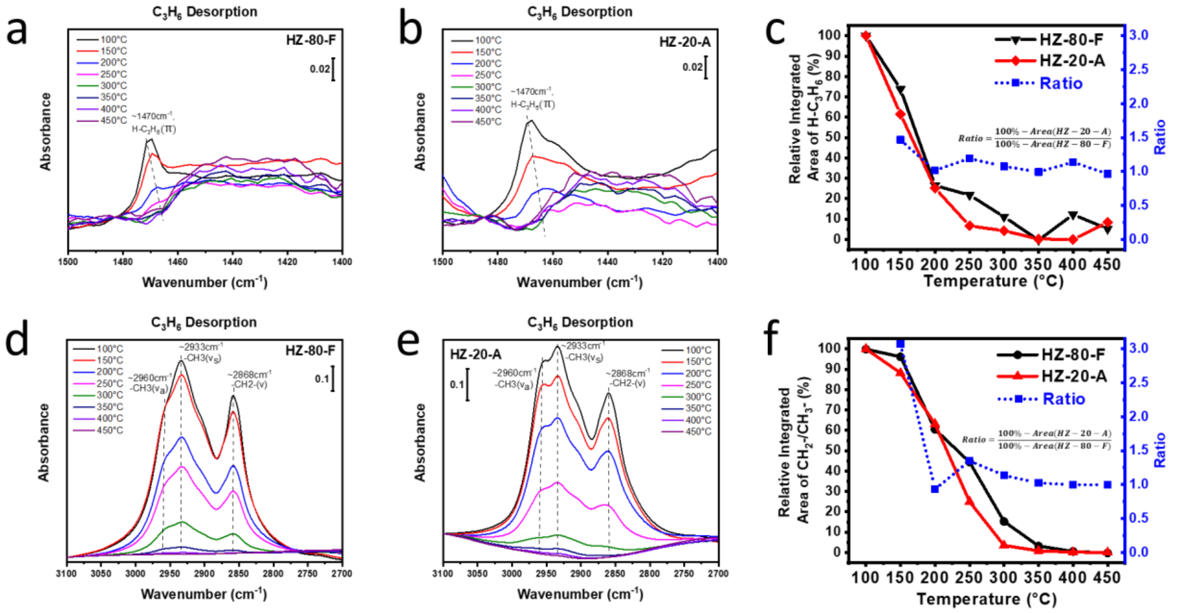

Supplementary Figure 10. ***In situ*** **DRIFTS spectra of two proton-exchanged ZSM-5 film samples during the propene desorption from 100 °C to 450 °C at 2 °C min^{-1} in 12 % O_2 / N_2. a, d** DRIFTS spectra at 1500-1400 and 3100-2700 cm^{-1} of proton-exchanged conventional ZSM-5 film (HZ-80-F), respectively. **b, e** DRIFTS spectra at 1500-1400 and 3100-2700 cm^{-1} of proton-exchanged array-structured ZSM-5 film (HZ-20-A), respectively. **c, f** The relative change of the integrated peak area of H-bonded C_3H_6 (1470 cm^{-1}) and CH_2-/CH_3- (3080-2750 cm^{-1}) from DRIFTS spectra during the propene desorption from 100 °C to 450 °C, respectively. Source data are provided as a Source Data file.

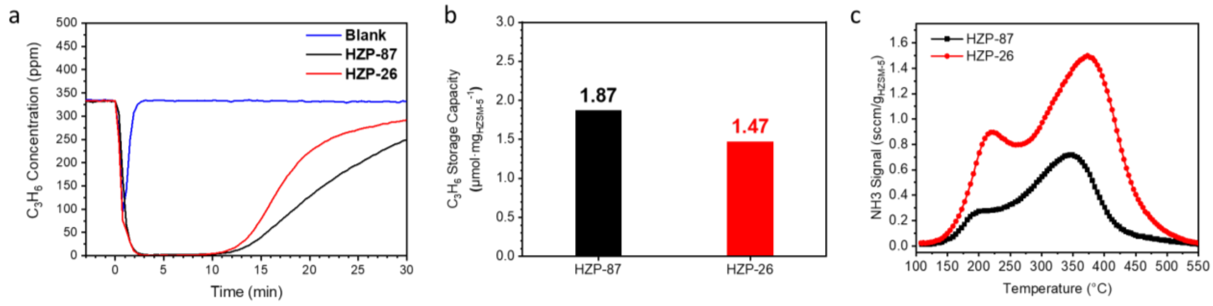

Supplementary Figure 11. Propene adsorption behavior of washcoated H-ZSM-5 samples (HZP-87 in black line and HZP-26 in red line) within 30 min adsorption. a Propene concentration change over the testing time. The result of blank cordierite substrate was included (blue line). **B** Calculated propene storage capacity after 30 min adsorption. **C** NH_3-TPD of HZP-87 and HZP-26. The adsorption testing was carried out at 100 °C under a gas mixture of 333 ppm C_3H_6 + 12 % O_2 / N_2 with a space velocity of ~24,000 h-1. Source data are provided as a Source Data file.

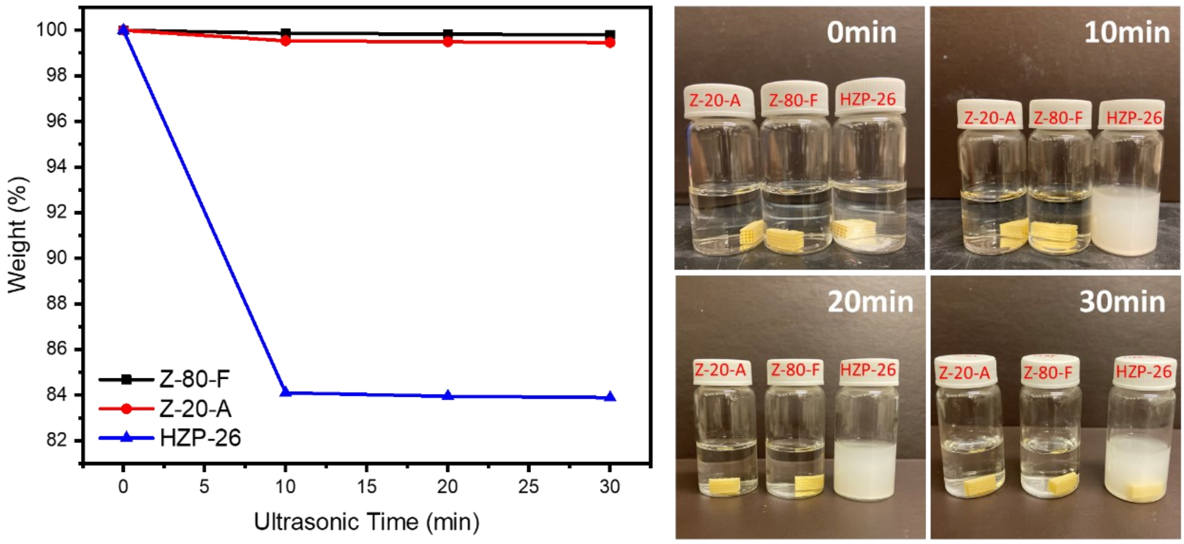

Supplementary Figure 12. Mechanical stability test for ZSM-5 film (Z-80-F, black line), ZSM-5 nanoarray (Z-20-A, red line), and ZSM-5 washcoated samples (HZP-26, blue line) by sonication in DI water for 30 min. Source data are provided as a Source Data file.

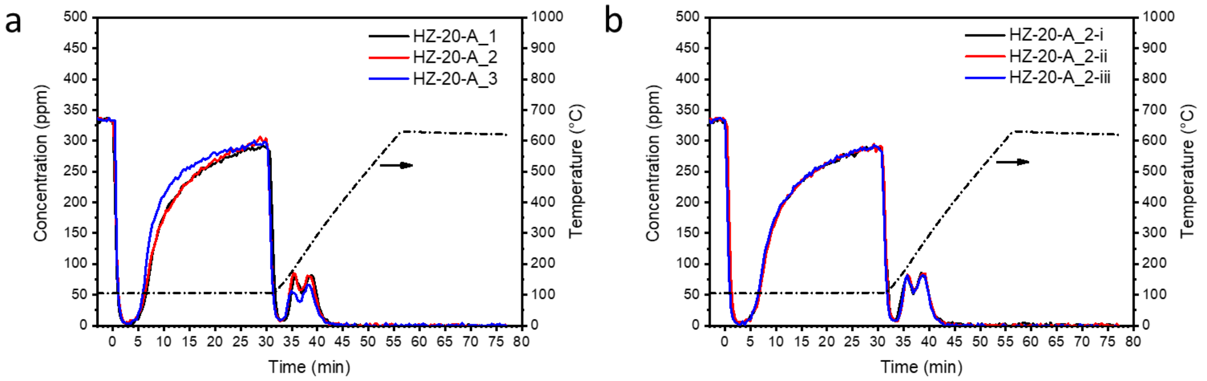

Supplementary Figure 13. Reproducibility of C_3H_6 adsorption in ZSM-5 nanoarray samples made by the identical synthesis processes. **a** Results from samples prepared in three batches. **b** Results from one sample in three back-to-back cyclic tests. Source data are provided as a Source Data file.

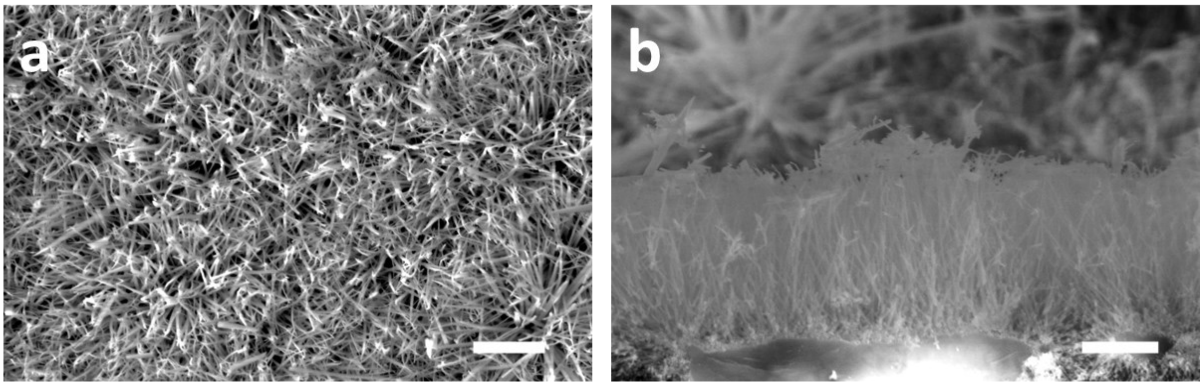

Supplementary Figure 14. Scanning electron microscope (SEM) images of Co_3O_4 nanoarray grown on the cordierite surface. **a** Top view. **b** Cross-sectional view. Scale bars: 5 μm.

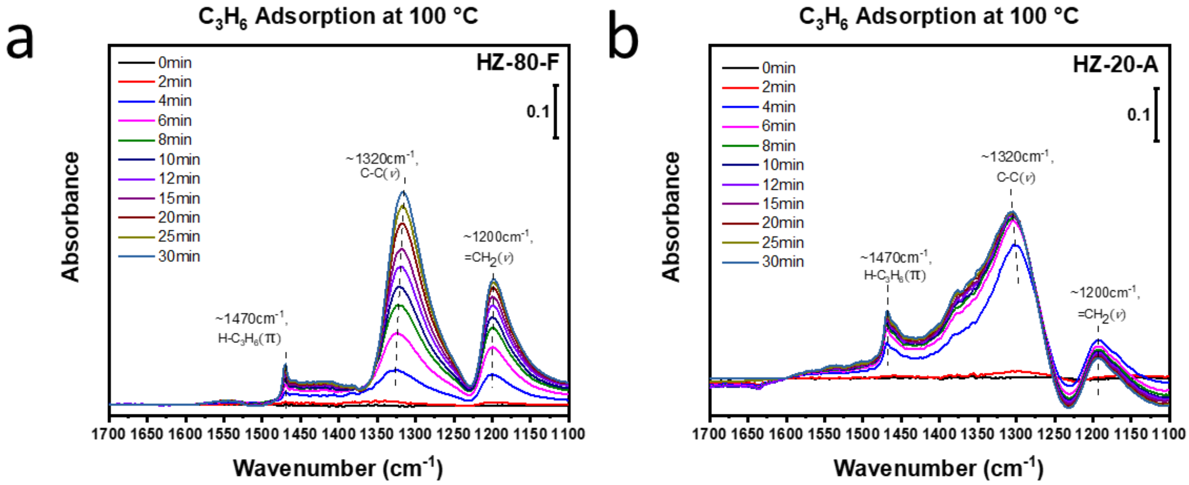

Supplementary Figure 15. *In situ* DRIFTS spectra of two proton-exchanged ZSM-5 film samples during the propene adsorption under 333 ppm C_3H_6 + 12 % O_2 / N_2 at 100 °C for 30 min at 1700-1100 cm^{-1}. a proton-exchanged conventional ZSM-5 film (HZ-80-F) and **b** proton-exchanged array-structured ZSM-5 film (HZ-20-A) during the propene adsorption. In these spectra, the C=C stretch peak from C_3H_6 around 1650 cm^{-1} is hardly to be observed, which is due to the low C_3H_6 partial pressure used (only 333 ppm C_3H_6 was fed into DRIFTS chamber[3]). Instead, C-C (~1320 cm^{-1}) and C-H (~1200 cm^{-1}) signals from the oligomerization products can be clearly viewed after 30 min propene feeding, indicating a strong oligomerization pathway. It is noted that the peak centered at 1470 cm^{-1} was used for further analysis as the non-conventional DRIFTS bands, at wavenumbers lower than 1400 cm^{-1}, for C_3H_6 might be impacted by the influence of ZSM-5 absorption.

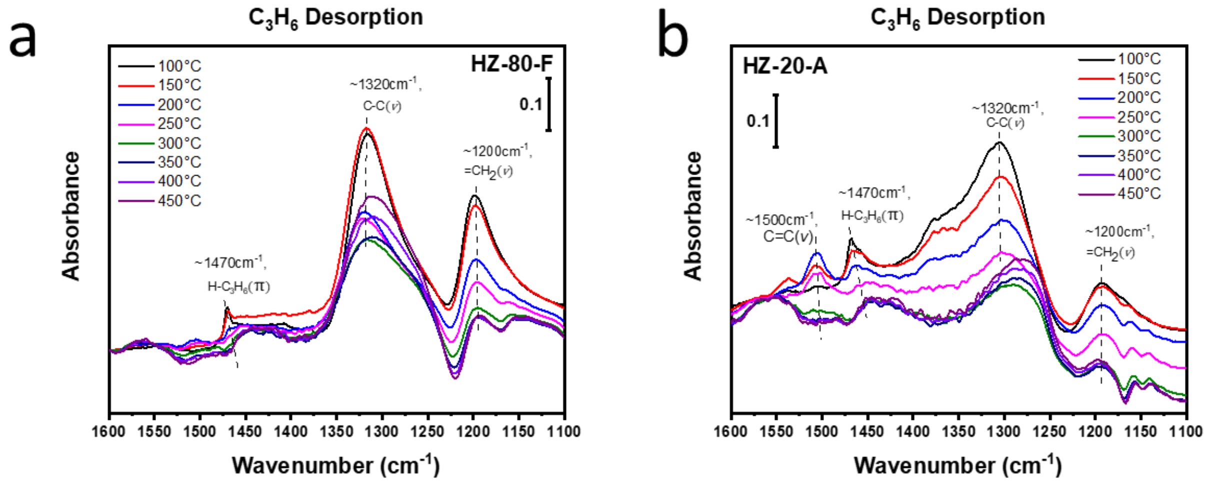

Supplementary Figure 16. *In situ* DRIFTS spectra at 1600-1100 cm^{-1} of two proton-exchanged ZSM-5 film samples during the propene desorption from 100 °C to 450 °C at 2 °C min^{-1} in 12 % O_2 / N_2. a proton-exchanged conventional ZSM-5 film (HZ-80-F) and **b** array-structured ZSM-5 film (HZ-20-A) during the propene desorption.

Supplementary Tables

Supplementary Table 1. Sample information for as-synthesized Z-80-F and Z-20-A.

Sample	Si/Al[a]	ZSM-5 Loading, wt.%	Crystallographic Preferred Orientation[b] $CPO([002]/([200]+[020]))$
Z-80-F	83.5	26.0	0.82
Z-20-A	15.6	19.2	0.87

[a] The atomic ratio of Si/Al is determined by the energy dispersive spectroscopy (EDS) that is combined with high-resolution transmission electron microscope (HRTEM).

[b] The value of crystallographic preferred orientation (CPO)[4, 5, 6] is calculated by: $CPO([X]/[Y]) = \frac{I_S^{[X]}/I_S^{[Y]} - I_P^{[X]}/I_P^{[Y]}}{I_S^{[X]}/I_S^{[Y]}}$, where $[X]$ and $[Y]$ represent the Miller indexes of the crystallographic planes, and I_S and I_P are the intensities from the ZSM-5 films on cordierite and commercial ZSM-5 powders. The value of $CPO([002]/([200]+[020]))$ would be closer to 1 if the preferential orientation of *c*-axis dominates in the film.

Supplementary Table 2. Quantitative summary of texture characteristics of the washcoated ZSM-5 samples that were dipped-coated with commercial powers at Si/Al = 87 (HZP-87) and Si/Al = 26 (HZP-26).

Characteristics	Unit	HZP-87	HZP-26
$S_{BET,\ Sample}$[a]	$m^2\ g^{-1}$	75	64
$S_{BET,\ ZSM\text{-}5}$[b]	$m^2\ g^{-1}$	333	278
$S_{BET,\ Cordierite}$[b]	$m^2\ g^{-1}$	0.004	0.004
$S_{Ext,\ ZSM\text{-}5}$[b]	$m^2\ g^{-1}$	159	127
$V_{total,\ Sample}$[a]	$cm^3\ g^{-1}$	0.049	0.051
$V_{meso,\ Sample}$[a]	$cm^3\ g^{-1}$	0.033	0.037

[a] Cordierite substrates were included for the calculation of sample's surface area ($S_{BET,Sample}$), total pore volume ($V_{total,Sample}$), and mesopore volume ($V_{meso,Sample}$) in the N_2 adsorption-desorption isothermal test.

[b] The surface area of ZSM-5 on the washcoated samples ($S_{BET,\ ZSM\text{-}5}$) was estimated by: $S_{BET,\,Sample} \times Mass_{Sample} = S_{BET,\,Cordierite} \times Mass_{Cordierite} + S_{BET,\,ZSM-5} \times Mass_{ZSM-5}$. Similarly, as cordierite substrate barely contributed to the sample's external surface area, assuming $S_{Ext,\,Cordierite} = 0$, the external surface area of ZSM-5 film ($S_{Ext,\ ZSM\text{-}5}$) could be calculated by: $S_{Ext,\,Sample} \times Mass_{Sample} = S_{Ext,\,ZSM-5} \times Mass_{ZSM-5}$.

Supplementary Table 3. Propene adsorption performance of ZSM-5 nanoarray samples for reproducibility demonstration*.

Category	Sample	Loading Ratio, wt.%	Zeolite Mass, mg	C_3H_6 Adsorption Capacity, μmol mg_{HZSM-5}^{-1}	
	HZ-20-A_1	17.6%	33.9	1.34	
Batch-to-batch	HZ-20-A_2	19.8%	36.9	1.42	(1.44±0.12)
	HZ-20-A_3	17.7%	32.3	1.57	
	HZ-20-A_2-i	19.8%	36.9	1.42	
Back-to-back Cyclic Test	HZ-20-A_2-ii	19.8%	36.9	1.41	(1.42±0.0.1)
	HZ-20-A_2-iii	19.8%	36.9	1.43	

* In order to demonstrate the reproducibility of our synthesis approach of ZSM-5 nanoarray samples, three batches following the same procedures were synthesized and denoted as HZ-20-A-1, HZ-20-A-2 and HZ-20-A-3. The propene adsorption performance was then conducted on these three samples and repeated on the same selected sample back-to-back for three times (HZ-20-A-2-i, HZ-20-A-2-ii, HZ-20-A-2-iii).

Supplemental References

1. Wong WC, Au LTY, Lau PPS, Ariso CT, Yeung KL. Effects of synthesis parameters on the zeolite membrane morphology. *Journal of Membrane Science* **193**, 141-161 (2001).

2. Bonilla G, Vlachos DG, Tsapatsis M. Simulations and experiments on the growth and microstructure of zeolite MFI films and membranes made by secondary growth. *Microporous and Mesoporous Materials* **42**, 191-203 (2001).

3. Rubeš M, Koudelková E, de Oliveira Ramos FS, Trachta M, Bludský O, Bulánek R. Experimental and Theoretical Study of Propene Adsorption on K-FER Zeolites: New Evidence of Bridged Complex Formation. *The Journal of Physical Chemistry C* **122**, 6128-6136 (2018).

4. Ulla MA, Mallada R, Coronas J, Gutierrez L, Miró E, Santamaría J. Synthesis and characterization of ZSM-5 coatings onto cordierite honeycomb supports. *Applied Catalysis A: General* **253**, 257-269 (2003).

5. Zamaro JM, Ulla MA, Miró EE. Growth of mordenite on monoliths by secondary synthesis. Effects of the substrate on the coating structure and catalytic activity. *Applied Catalysis A: General* **314**, 101-113 (2006).

6. Wu A, Tang C, Zhong S, Wang B, Zhou J, Zhou R. Synthesis optimization of (h 0 h)-oriented silicalite-1 membranes for butane isomer separation. *Separation and Purification Technology* **214**, 51-60 (2019).
